# Feature importance correlation from machine learning indicates functional relationships between proteins and similar compound binding characteristics

**DOI:** 10.1038/s41598-021-93771-y

**Published:** 2021-07-09

**Authors:** Raquel Rodríguez-Pérez, Jürgen Bajorath

**Affiliations:** 1 Department of Life Science Informatics, B-IT, LIMES Program Unit Chemical Biology and Medicinal Chemistry, Rheinische Friedrich-Wilhelms-Universität, Friedrich-Hirzebruch-Allee 6, 53115 Bonn, Germany; 2 Novartis Institutes for Biomedical Research, Novartis Campus, 4002 Basel, Switzerland

**Keywords:** Computational biology and bioinformatics, Drug discovery, Cheminformatics

## Abstract

Machine learning is widely applied in drug discovery research to predict molecular properties and aid in the identification of active compounds. Herein, we introduce a new approach that uses model-internal information from compound activity predictions to uncover relationships between target proteins. On the basis of a large-scale analysis generating and comparing machine learning models for more than 200 proteins, feature importance correlation analysis is shown to detect similar compound binding characteristics. Furthermore, rather unexpectedly, the analysis also reveals functional relationships between proteins that are independent of active compounds and binding characteristics. Feature importance correlation analysis does not depend on specific representations, algorithms, or metrics and is generally applicable as long as predictive models can be derived. Moreover, the approach does not require or involve explainable or interpretable machine learning, but only access to feature weights or importance values. On the basis of our findings, the approach represents a new facet of machine learning in drug discovery with potential for practical applications.

## Introduction

In medicinal chemistry and drug design, machine learning (ML) has long been applied to predict molecular properties of compounds, especially biological activity^1,2^. ML models can be developed to qualitatively or quantitatively predict compound activity against given biological targets. For early compound prioritization, classification models derived to distinguish between specifically active and inactive compounds are preferentially used^3,4^. In a given chemical reference space defined by selected molecular representations (descriptors), such models are trained to correlate chemical/structural patterns with biological activity and predict new active compounds. Furthermore, in compound optimization, regression models are often used to predict numerical compound potency values^4,5^. In this case, feature patterns of active compounds are correlated with potency values of known compounds to enable quantitative predictions. In general, ML classification or regression models can be derived to predict any other physicochemical or biological compound properties.

The qualitative and quantitative ML prediction strategies outlined above apply regardless of the algorithms that are used and their computational complexity. Given the rising popularity of deep neural network architectures in many scientific fields including medicinal chemistry and drug design^6–8^, there is an intense debate concerning explainable and interpretable ML^9–11^. In the practice of medicinal chemistry, the acceptance of “black box” ML predictions is typically low. Simply put, chemists are reluctant to synthesize and test compounds resulting from predictions they do not understand; rightly so. Of course, the confidence in black box predictions is not only low in medicinal chemistry and drug design, but also in many other fields, which in part results from the use of models that are too complex for the prediction tasks at hand^10^. Regardless, various efforts have been made to aid in explaining ML predictions^11^ including feature weighting and mapping^12,13^. These techniques aim to identify structural features that determine predictions. Mapping such features onto test compounds helps to analyze the results from a chemical perspective. Accordingly, estimation of feature weights or importance values is applicable to better understand non-transparent ML model components that drive predictions.

However, while feature weighting has thus far been used to aid in model interpretation, albeit only in a limited number of studies, it can also be adapted to provide the basis for an approach that is completely distinct from model explanations, as reported herein. We have reasoned that feature importance distributions might be determined as a model-agnostic and model-internal computational signature of data set properties, without any requirements to interpret predictions. To these ends, we have further extended the feature weighting approach and introduce feature importance correlation analysis to reveal similar data set signatures. In our proof-of-concept study, the methodology was applied to compound activity prediction models where high feature importance correlation served as an indicator of similar compound binding characteristics of proteins as well as functional relationships. To our knowledge, feature importance correlation analysis represents a novel concept in ML and computer-aided drug discovery. In the following, the results of our proof-of-concept investigation are presented.

## Results

### Analysis strategy

To investigate feature importance correlation between models for compound activity prediction on a large scale, we prioritized target proteins from different classes. In each case, at least 60 compounds from different chemical series with confirmed activity against a given protein and available high-quality activity data were required for training and testing (positive instances) and the resulting predictions had to reach reasonable to high accuracy (see “Methods”). As negative training and test instances, compounds without known biological activity from medicinal chemistry vendors were randomly selected. For feature importance correlation analysis, the negative class should ideally provide a consistent inactive reference state for all activity predictions. For the widely distributed targets with high-confidence activity data studied here, such experimentally confirmed consistently inactive compounds are unavailable, at least in the public domain. Therefore, the negative (inactive) class was represented by a consistently used random sample of compounds without biological annotations (see “Methods”). All active and inactive compounds were represented using a topological fingerprint calculated from molecular structure. To ensure generality of feature importance correlation and establish proof-of-concept, it was important that a chosen molecular representation did not include target information, pharmacophore patterns, or features prioritized for ligand binding.

For classification, the random forest (RF) algorithm was applied as a widely used standard in the field, due to its suitability for high-throughput modeling and the absence of non-transparent optimization procedures. Feature importance was assessed adapting the Gini impurity criterion (see “Methods”), which is well-suited to quantify the quality of node splits along decision tree structures (and also inexpensive to calculate). Feature importance correlation was determined using Pearson and Spearman correlation coefficients (see “Methods”), which account for linear correlation between two data distributions and rank correlation, respectively. For our proof-of-concept study, the ML system and calculation set-up was made as transparent and straightforward as possible, preferably applying established standards in the field.

### Classification performance

A total of 218 qualifying proteins were selected covering a wide range of pharmaceutical targets, as summarized in Supplementary Table S1. Target protein selection was determined by requiring sufficient numbers of active compounds for meaningful ML while applying stringent activity data confidence and selection criteria (see “Methods”). For each of the corresponding compound activity classes, a RF model was generated. The model was required to reach at least a compound recall of 65%, Matthew’s correlation coefficient (MCC) of 0.5, and balanced accuracy (BA) of 70% (otherwise, the target protein was disregarded). Table 1 reports the global performance of the models for the 218 proteins in distinguishing between active and inactive compounds. The mean prediction accuracy of these models was above 90% on the basis of different performance measures. Hence, model accuracy was generally high (supported by the use of negative training and test instances without bioactivity annotations), thus providing a sound basis for feature importance correlation analysis.

**Table 1:** Model performance.

	Recall	BA	F1	MCC
Mean	93%	96%	0.90	0.90
Std	8%	4%	0.12	0.11
Min	66%	83%	0.47	0.54

The mean, standard deviation (Std) and minimum (Min) values are reported for multiple metrics including recall, BA, F1 score, and MCC across the 218 RF models.

### Feature importance analysis

Contributions of individual features to correct activity predictions were quantified. The specific nature of the features depends on chosen molecular representations. Here, each training and test compound was represented by a binary feature vector of constant length of 1024 bits (see “Methods”). Each bit represented a topological feature. For RF-based activity prediction, sequential feature combinations maximizing classification accuracy were determined. As detailed in the Methods, for recursive partitioning, Gini impurity at nodes (feature-based decision points) was calculated to prioritize features responsible for correct predictions. For a given feature, Gini importance is equivalent to the mean decrease in Gini impurity calculated as the normalized sum of all impurity decrease values for nodes in the tree ensemble where decisions are based on that feature. Thus, increasing Gini importance values indicate increasing relevance of the corresponding features for the RF model. Gini feature importance values were systematically calculated for all 218 target-based RF models. On the basis of these values, features were ranked according their contributions to the prediction accuracy of each model.

### Feature importance correlation

**Figure 1:**
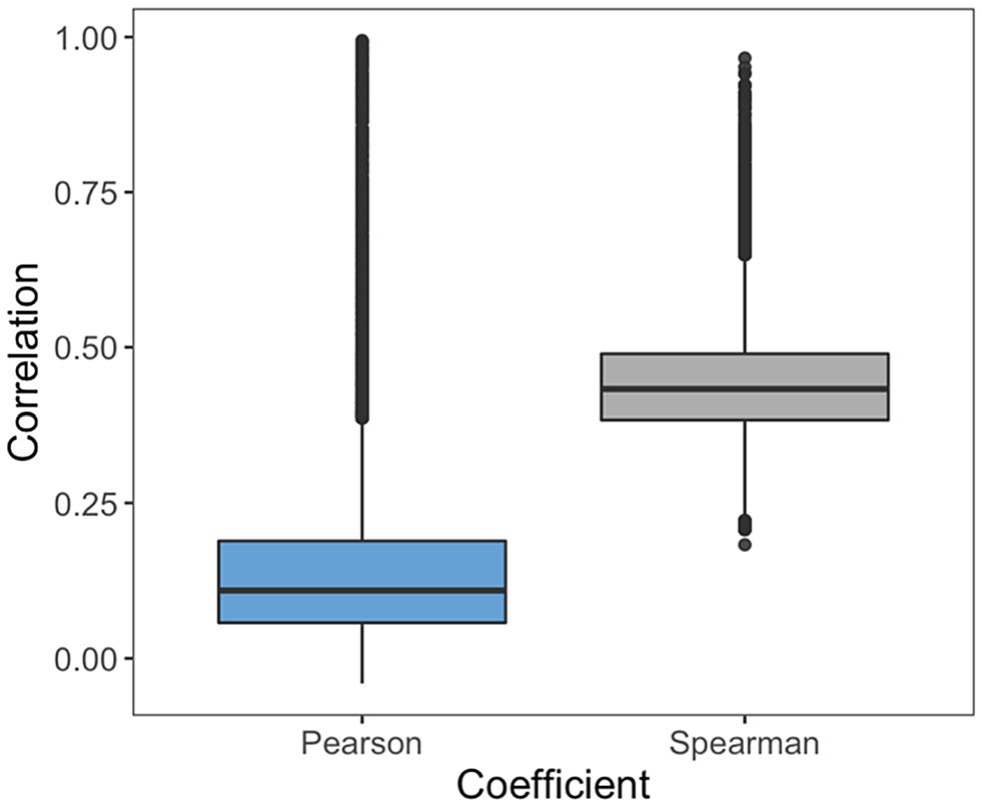
Feature importance correlation. Distributions of feature importance correlation values are reported in boxplots for all protein pairs in the data set. Correlation values were calculated using the Pearson (blue) and Spearman (gray) coefficients.

A compound activity class implicitly captures ligand binding characteristics for a given target. Accordingly, we hypothesized that the feature importance ranking derived from a target-based RF model might represent a computational signature of binding characteristics of this target. If so, feature correlation calculated on the basis of these rankings could be used as an indicator for relationships between targets and their binding characteristics. Of note, a feature ranking captures model-internal information without taking any target criteria into account. This has important implications for feature importance correlation. If accurate prediction models can be derived, as in this case, neither the chemical nature of the features, nor their encoding needs to be further evaluated. Instead, only their correlation (or similarity) must be determined. Therefore, following our approach, a critically important step was determining whether feature importance correlation differed among protein pairs as a potential indicator of varying relationships. Figure 1 shows the distribution of systematically calculated Pearson and Spearman correlation coefficients for comparison of feature importance values and feature rankings, respectively. For both coefficients, a large value range was observed. As anticipated for diverse target proteins, many comparisons revealed weak correlation, with median coefficient values of 0.11 and 0.43, respectively. However, there were numerous “statistical outliers” with larger values, in part indicating strong correlation. Supplementary Fig. S1 shows a heatmap capturing all 47,524 pairwise comparisons that further illustrates these observations. In the map, target-based models were hierarchically clustered, revealing the formation of clusters by models with high feature importance correlation along the diagonal and the presence of varying degrees of correlation across the map. Hence, feature importance correlation analysis yielded different results warranting further investigation.

### Similar binding characteristics

**Figure 2:**
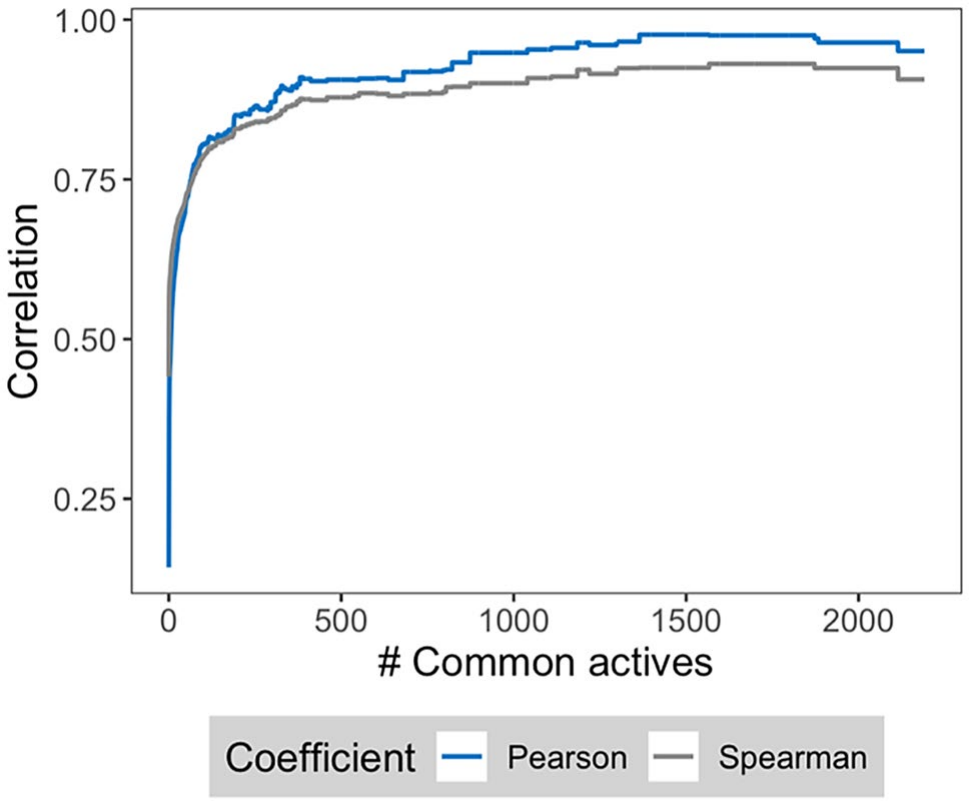
Correlation for protein pairs with common active compounds. Mean feature importance correlation values are reported for protein pairs with increasing numbers of shared compounds.

The next task was to determine whether strong feature importance correlation was indeed an indicator of related ligand binding characteristics. By definition, proteins sharing active compounds have similar binding characteristics. Therefore, we searched for pairs of targets with common ligands. While proteins forming 22,008 pairs (93%) did not have any active compounds in common, 452 protein pairs were found to share a single active compound, 527 pairs shared two to 10 actives, and 666 pairs more than 10 actives (with a maximum of 2191). Figure 2 reports the mean feature importance correlation for protein pairs sharing increasing numbers of active compounds and reveals a clear relationship. In the presence of shared actives, correlation was generally strong and further increasing with increasing numbers of common compounds. Thus, these findings clearly showed that feature importance correlation revealed similar binding characteristics. We also hierarchically clustered proteins from pairs with strong correlation. Supplementary Fig. S2 shows a heatmap for a subset of proteins from pairs with a Pearson coefficient of at least 0.5. This subset resulted from hierarchical clustering of the data sets based on pairwise correlation coefficient values and represented the largest cluster, which was enriched with G protein coupled receptors. In this heatmap, proteins from the same enzyme or receptor families were grouped together. Members of the same family typically shared a few active compounds.

### Functional relationships

In light of these findings, we then asked the question whether feature importance correlation might also serve as an indicator of functional relationships between proteins that are independent of active compounds. While this supposition appeared to be far-fetched, we devised an analysis scheme for investigating it. Therefore, Gene Ontology (GO) terms covering cellular component, molecular function, and biological process were extracted for the 218 proteins. Between four and 189 GO terms were obtained per protein (with a mean of 43). For each protein pair, we then calculated the Tanimoto coefficient (Tc) to quantify the overlap in GO terms:

$$\mathrm{T}\mathrm{c} \left(\mathrm{A},\mathrm{B}\right)= \frac{\left|\mathrm{A} \cap \mathrm{B}\right|}{\left|\mathrm{A}\cup \mathrm{B}\right|}$$

Here, *A* and *B* represent the sets of GO terms for a pair of proteins A and B, respectively.

Accordingly, GO Tc values served as a measure of functional relatedness of proteins from a pair. Only 2058 protein pairs (8.7%) did not share any GO terms (resulting in Tc = 0). Supplementary Fig. S3 reports the global distribution of GO Tc values over all the 218 proteins. The distribution is Gaussian-like on a logarithmic scale, as expected in the presence of many random values. The mode of the distribution maps to small Tc value < 0.10.

**Figure 3:**
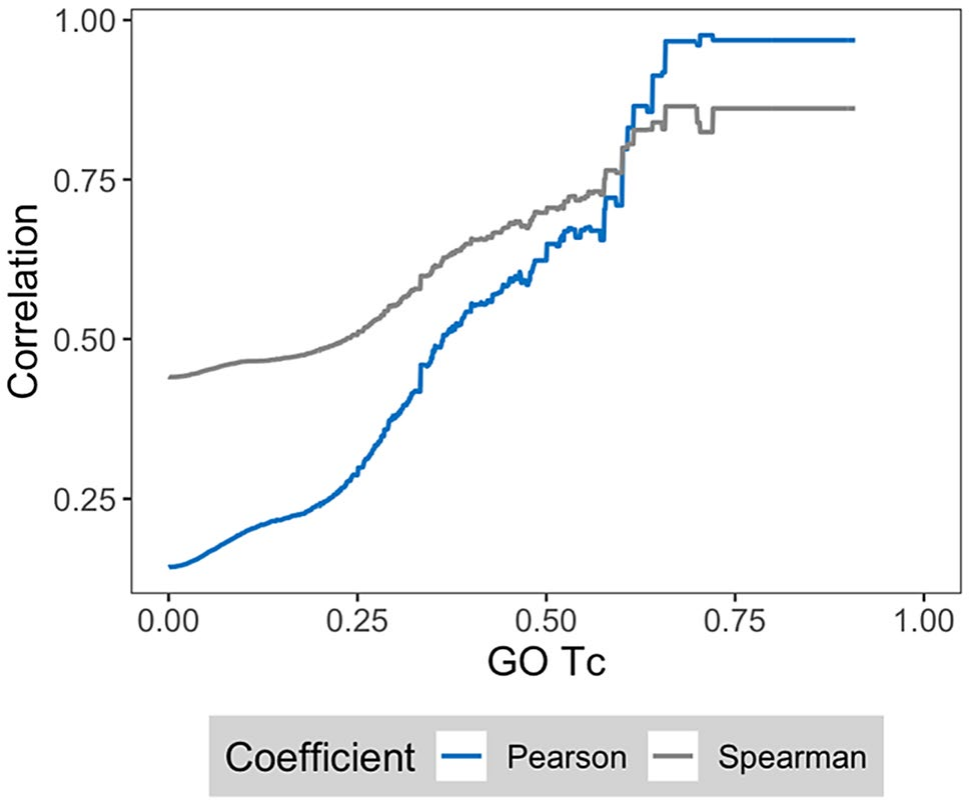
Correlation for protein pairs with common GO annotations. Mean feature importance correlation values are reported for protein pairs with increasing GO Tc values.

Figure 3 shows feature importance correlation for protein pairs with increasing GO Tc values, which reveals another clear relationship. Correlation increased with increasing functional relatedness of paired proteins. At GO Tc values of ~ 0.75, maximal correlation was observed. Given the global GO Tc distribution, values of 0.50 or greater were of high significance. Thus, feature importance correlation also was a strong indicator of functional relationships not taking compound information into account.

### From correlations to multi-target compounds

**Figure 4:**
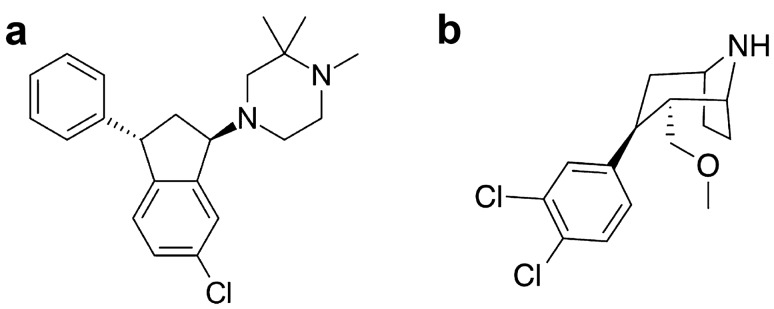
Multi-target compounds. Shown are two exemplary clinical compounds with different activity. Each of these compounds is active against strongly correlated target proteins. (**a**) Zicronapine, with activity against HTR2A and dopamine D1 and D2 receptors. (**b**) Serotonin-norepinephrine-dopamine reuptake inhibitor with activity against the dopamine, norepinephrine, and serotonin transporter proteins.

Since feature importance correlation indicates functional relationships between proteins as well as similar binding characteristics, high correlation might also imply that proteins share ligands, as analyzed above. Figure 4 shows two exemplary compounds with activity against different targets that were identified based on this premise. For example, the serotonin 5-HT 2A receptor (HTR2A) was strongly correlated with several dopamine receptor isoforms (correlation coefficients of 0.90 or above). We searched the therapeutic target database (TTD)^14^ for new drug candidates with corresponding target annotations, revealing zicronapine (currently in phase III clinical trials), which modulates HTRA2 and the dopamine D1 and D2 receptors (Fig. 4a). Another exemplary set of proteins with strong pairwise correlation included the dopamine, norepinephrine, and serotonin transporter proteins. In this case, TTD was found to contain a serotonin-norepinephrine-dopamine reuptake inhibitor with reported activity against these three targets (Fig. 4b). In light of such examples, it is conceivable that feature importance correlation could also be used to suggest new targets for known bioactive compounds. For example, if two proteins display strong feature importance correlation, known active compounds might be subjected to cross-testing.

### Unexpected correlations

Supplementary Fig. S4 shows that proteins forming pairs with increasing feature importance correlation tended to originate from the same target group. Hence, many of these paired proteins were related, as one might expect. However, strongly correlated pairs also contained distinct proteins. While correlation coefficient values larger than 0.75 were mostly observed for proteins from the same class, values of strongly correlated protein pairs from different classes fell mostly into the range from 0.50 to 0.75. Table 2 reports examples of strongly correlated pairs of proteins from different target groups (which were among the statistical outliers in Fig. 1). Although proteins in these pairs did not share any active compounds and were virtually unrelated, strong feature importance correlation indicated (non-obvious) functional relationships. For example, with a Pearson correlation coefficient of 0.77, strong correlation was observed for cystinyl aminopeptidase and estrogen receptor alpha and beta (a protease and nuclear hormone receptors, respectively). These proteins had only five GO terms in common (signal transduction, zinc ion binding, metal ion binding, cell–cell signaling, and protein binding) that were not indicative of a specific relationship. Nonetheless, there was a physiological link between these proteins. Cystinyl aminopeptidase cleaves vasopressin, oxytocin, and other peptide hormones and catalyzes the final step in the conversion of angiotensinogen to angiotensin IV. During pregnancy, it is secreted into the maternal serum. Estrogen receptor alpha and beta also trigger degradation of peptide hormones such as vasopressin, oxytocin, or angiotensin III and aid in homeostasis during pregnancy^15^. Feature importance correlation indicated a number of other non-obvious relationships (Table 2), for example, between adenosine A1 receptor and the PI3-kinase p110-delta subunit. Of note, other G protein coupled receptors and protein kinases are known to share ligands, further supporting relationships between these different protein classes.

**Table 2:** Exemplary strongly correlated pairs of proteins from different classes.

Target 1		Target 2		Pearson/Spearman correlation
Name	Classification	Name	Classification	
Cystinyl aminopeptidase	Enzyme/protease	Estrogen receptor beta	Transcription factor/nuclear receptor	0.77/0.37
Cystinyl aminopeptidase	Enzyme/protease	Estrogen receptor alpha	Transcription factor/nuclear receptor	0.72/0.41
Corticotropin releasing factor receptor 1	Membrane receptor/G protein coupled receptor (GPCR)	Phosphodiesterase 10A	Enzyme/hydrolase	0.72/0.41
Adenosine A1 receptor	Membrane receptor/GPCR	PI3-kinase p110-delta subunit	Enzyme/transferase	0.65/0.49
Carboxyl-esterase 2	Enzyme	Neuronal acetylcholine receptor protein alpha-7 subunit	Ion channel/ligand-gated ion channel	0.61/0.50
Prostanoid DP receptor	Membrane receptor/GPCR	Protein-tyrosine phosphatase 1B	Enzyme/hydrolase	0.60/0.45
Adenosine A2a receptor	Enzyme/hydrolase	Phosphodiesterase 10A	Membrane receptor/GPCR	0.60/0.44
Monoamine oxidase B	Enzyme/oxidoreductase	Serotonin 2c receptor	Membrane receptor/GPCR	0.59/0.63
Carbonic anhydrase IX	Enzyme/lyase	Serotonin 6 (5-HT6) receptor	Membrane receptor/GPCR	0.58/0.65
Peroxisome proliferator-activated receptor gamma	Transcription factor/nuclear receptor	Protein-tyrosine phosphatase 1B	Enzyme/hydrolase	0.57/0.49
Beta amyloid A4 protein	Membrane receptor	Serotonin transporter	Transporter/eletrochemical transporter	0.53/0.57
Acyl coenzyme A: cholesterol acyltransferase	Enzyme	Cannabinoid CB1 receptor	Membrane receptor/GPCR	0.36/0.69

## Conclusion

In drug discovery research, ML is mostly applied to predict molecular properties and search for active compounds. Among practitioners, there is often limited trust in computational predictions if they cannot be rationalized from a chemical or biological perspective. To alleviate the black box character of many (but not all) ML models, techniques such as feature weighting and mapping can be applied to help explain the predictions. Such efforts are typically focused on individual models and their output. In this work, we have investigated feature importance on a large scale for a purpose completely distinct from model interpretation. The concept of *feature importance correlation* introduced in this study aims to identify relationships between proteins on the basis of ML model-internal information without the need to explain individual predictions. Instead, computational signatures of feature importance are generated and feature importance correlation is quantified. Therefore, we have systematically determined feature importance for compound activity prediction models of 218 target proteins and assessed feature importance correlation in a pairwise manner. The underlying idea was that correlation between important features learned by independent models for different target proteins should be an indicator of relationships between these proteins. Accordingly, in the case of compound activity predictions, strong correlation should indicate similar binding characteristics of paired proteins. Proof-of-concept for this conjecture was provided by confirming a high degree of feature importance correlation for models of proteins that shared active compounds and thus had similar binding characteristics. Moreover, we also found that strong feature importance correlation was an indicator of functional relationships between proteins according to GO, not taking active compounds into account; a surprising finding. These results also indicate that populations of active compounds of target proteins implicitly capture more functionally relevant information than one might expected.

From an ML perspective, major conditions of feature importance correlation analysis represent an attractive aspect of this concept. Individual models must be consistently derived and sufficiently accurate and discrete features must contribute to the predictions. As long as these requirements are met, the nature of the features and the specifics of the ML algorithm may vary, hence alleviating any need for model interpretation. Accordingly, a variety of molecular representations, ML methods, and metrics are applicable to determine feature importance correlation or similarity. These include approaches such as RF and the Gini importance measure that are computational efficient and suitable for large-scale analysis, as reported herein.

Strong feature importance correlation was also observed for a subset of proteins from different target groups that did not share active compounds. Such unexpected relationships are of particular interest from a functional viewpoint and also of practical relevance for applications such as drug repurposing. New targets for existing drugs are typically inferred from binding site or ligand similarity, but have not been predicted on the basis of global binding characteristics or functional relationships. Importantly, feature importance correlation analysis does neither require the comparison of compounds with activity against different targets, nor the detection of multi-target compounds, but establishes higher-level relationships between target proteins of interest.

In summary, in light of the findings reported herein, the concept of feature importance correlation represents a new facet of ML in drug discovery research and provides new opportunities. For example, one can further explore unexpected relationships between target proteins revealed by feature importance correlation.

## Methods

### Compound data and features

Compound activity classes were assembled from ChEMBL^16^ and negative training and test compounds without target annotations were randomly selected from the ZINC database^17^ comprising compounds from medicinal chemistry vendors. There was no overlap between the selected ChEMBL and ZINC compounds. For feature importance analysis, it must be ensured that the negative class represents an inert reference state for the predictions. Since ChEMBL (or other public database) do not contain confirmed consistently inactive compounds for the qualifying proteins, this was best accomplished by a random sample of compounds without biological annotations.

From ChEMBL, active compounds were selected on the basis of stringent criteria. Specifically, compounds were only considered if high-confidence activity data were available, i.e. K_i_ values from highest-confidence (score 9) direct interaction assays with individual human proteins. Compounds with multiple measurements not falling into the same order of magnitude were disregarded as well as borderline active compounds (pKi < 5). Activity classes were required to contain at least 60 compounds and two different chemical series, which were systematically determined using an algorithm for the identification of analog series^18^.

On the basis of these selection criteria, 218 compound data sets were assembled with activity against diverse target proteins, containing 62 to 3541 compounds per set. Proteins were assigned to target groups according the ChEMBL classification scheme^16^. Notably, there was only very limited compound overlap between different target-based data sets. Only 1,645 (7%) of all possible pairs shared active compounds.

Compounds were represented using the binary version of the extended connectivity fingerprint of diameter 4 (ECFP4) with a fixed length of 1024 feature bits^19^. This EFCP4 encoding was selected since it yielded accurate models and was of lower dimensionality than other hashed encodings, which was preferable for feature importance correlation analysis. The topological fingerprint was calculated with a Python-based in-house implementation from the *OpenEye OEChem toolkit*^20^.

### Machine learning

RF represents an ensemble of decision trees built in parallel using recursive partitioning^21^. For activity predictions, compounds are recursively divided into subsets to arrive at terminal nodes combining compounds with the same class label (active/inactive). To reduce high correlation among trees, models are trained on a bootstrap sample and a random subset of features are considered for node splitting (known as feature bagging)^22^. RF model predictions result from a consensus decision (majority vote) over the ensemble (set to 500 trees).

Two-fold cross validation was implemented to optimize the values of four model hyper-parameters via grid search including minimum samples per leaf node (candidate values: 1, 5, 10) minimum samples per split node (2, 8, 16), maximum number of features during node splitting (square root or logarithm to the base 2 of the number of features), and class weights. The latter parameter determined whether or not weighting was applied to compounds during training (inversely proportional to the class frequencies for imbalanced data sets). Decision trees were permitted to grow until leaves contained less than the minimum number of compounds required to split a node and the resulting branches had at least the minimum terminal node size. All RF calculations were carried out with *scikit-learn*^23^.

Activity classes were divided into training (75%) and test sets (25%) based on chemical series splitting^24^, and the same sets of negative training (1000) and test (10,000) instances were used for all models.

Model performance was evaluated on the basis of multiple performance measures including balanced accuracy (BA)^25^, F1 score^26^, and Matthew’s correlation coefficient (MCC)^27^.

$$\mathrm{r}\mathrm{e}\mathrm{c}\mathrm{a}\mathrm{l}\mathrm{l}=\frac{ \mathrm{T}\mathrm{P}}{\mathrm{T}\mathrm{P}+\mathrm{F}\mathrm{N}}$$

$$\mathrm{p}\mathrm{r}\mathrm{e}\mathrm{c}\mathrm{i}\mathrm{s}\mathrm{i}\mathrm{o}\mathrm{n}= \frac{ \mathrm{T}\mathrm{P}}{\mathrm{T}\mathrm{P}+\mathrm{F}\mathrm{P}}$$

$$\mathrm{B}\mathrm{A}=\frac{1}{2} \cdot \left(\frac{ \mathrm{T}\mathrm{P}}{\mathrm{T}\mathrm{P}+\mathrm{F}\mathrm{N}}+\frac{ \mathrm{T}\mathrm{N}}{\mathrm{T}\mathrm{N}+\mathrm{F}\mathrm{P}}\right)$$

$$\mathrm{F}1=2\cdot \frac{\mathrm{p}\mathrm{r}\mathrm{e}\mathrm{c}\mathrm{i}\mathrm{s}\mathrm{i}\mathrm{o}\mathrm{n}\cdot\mathrm{r}\mathrm{e}\mathrm{c}\mathrm{a}\mathrm{l}\mathrm{l}}{\mathrm{p}\mathrm{r}\mathrm{e}\mathrm{c}\mathrm{i}\mathrm{s}\mathrm{i}\mathrm{o}\mathrm{n}+\mathrm{r}\mathrm{e}\mathrm{c}\mathrm{a}\mathrm{l}\mathrm{l}}$$

$$\mathrm{M}\mathrm{C}\mathrm{C}=\frac{\mathrm{T}\mathrm{P}\times \mathrm{T}\mathrm{N}-\mathrm{F}\mathrm{P}\times \mathrm{F}\mathrm{N}}{\sqrt{(\mathrm{T}\mathrm{P}+\mathrm{F}\mathrm{P})(\mathrm{T}\mathrm{P}+\mathrm{F}\mathrm{N})(\mathrm{T}\mathrm{N}+\mathrm{F}\mathrm{P})(\mathrm{T}\mathrm{N}+\mathrm{F}\mathrm{N})}}$$

TP: true positive, TN: true negative, FP: false positive, FN: false negative.

### Feature importance correlation

The Gini impurity criterion^28^ was used as a measure of node-based recursive partitioning quality. Gini impurity is a metric from information theory defined as:

$$\mathrm{G}\mathrm{i}\mathrm{n}\mathrm{i} \, \mathrm{i}\mathrm{m}\mathrm{p}\mathrm{u}\mathrm{r}\mathrm{i}\mathrm{t}\mathrm{y}=\sum _{i=1}^{n}{p}_{i}(1-{p}_{i})$$

Here, ${p}_{i}$ is the frequency for class *i* at a given node, and *n* is 2 for binary classification. Accordingly, *Gini importance* for a given feature is equivalent to the mean decrease in Gini impurity, i.e. the normalized sum of all impurity decrease values for nodes in the RF where splitting was based on that feature^29^. Thus, increasing values indicate increasing feature importance for the RF model^29^.

Correlation or statistical association across feature importance values from the 218 RF models was computed using the Pearson and Spearman correlation coefficients. Pearson’s coefficient accounts for a proportional relationship whereas the Spearman coefficient quantifies rank correlation^30,31^. Both coefficients range from -1 to 1, accounting for perfect negative and positive correlation, respectively. Calculations were carried out with R and Python.

## Supplementary Information

Supplementary Information.

## Data Availability

Most calculations were carried out with public domain data and programs. The *OpenEye OEChem toolkit* requires a license from OpenEye Scientific Software, Inc.
