# Supplementary material for: Feature importance correlation from machine learning indicates functional relationships between proteins and similar compound binding characteristics
Source: Sci Rep. 2021 Jul 9;11:14245. doi: 10.1038/s41598-021-93771-y (PMC8270985; doi:10.1038/s41598-021-93771-y)
Supplement: Supplementary file 1 — Supplementary Information. [file 41598_2021_93771_MOESM1_ESM.docx]

**Supplementary Information**

**Feature importance correlation from machine learning indicates functional relationships between proteins and similar compound binding characteristics**

Raquel Rodríguez-Pérez^1,2^ and Jürgen Bajorath^1^*

^1^Department of Life Science Informatics, B-IT, LIMES Program Unit Chemical Biology and Medicinal Chemistry, Rheinische Friedrich-Wilhelms-Universität, Friedrich-Hirzebruch-Allee 6, D-53115 Bonn, Germany.

^2^Novartis Institutes for Biomedical Research, Novartis Campus, CH-4002 Basel

*Corresponding author

Tel: +49-228-73-69100, Fax: +49-228-73-69101, E-mail: [bajorath@bit.uni-bonn.de](mailto:bajorath@bit.uni-bonn.de)

**Supplementary Results**

*Supplementary Table S1*

*Supplementary Figures S1-S4*

**Supplementary Results**

**Table S1. Protein classification.** Reported are the numbers of proteins assigned to different target groups on the basis of the L1 and L2 ChEMBL classification scheme.

| **L1** | | **L2** | |
| --- | --- | --- | --- |
| **Group** | **# Proteins** | **Group** | **# Proteins** |
| Enzyme | 97 | G protein-coupled receptor | 85 |
| Membrane receptor | 91 | Protease | 37 |
| Ion channel | 8 | Other/missing | 33 |
| Other cytosolic protein | 6 | Kinase | 26 |
| Transcription factor | 6 | Lyase | 9 |
| Transporter | 5 | Transferase | 7 |
| Secreted protein | 2 | Nuclear receptor | 6 |
| Other nuclear protein | 1 | Oxidoreductase | 6 |
| Epigenetic regulator | 1 | Ligand-gated ion channel | 5 |
| Unclassified protein | 1 | Electrochemical transporter | 4 |

**
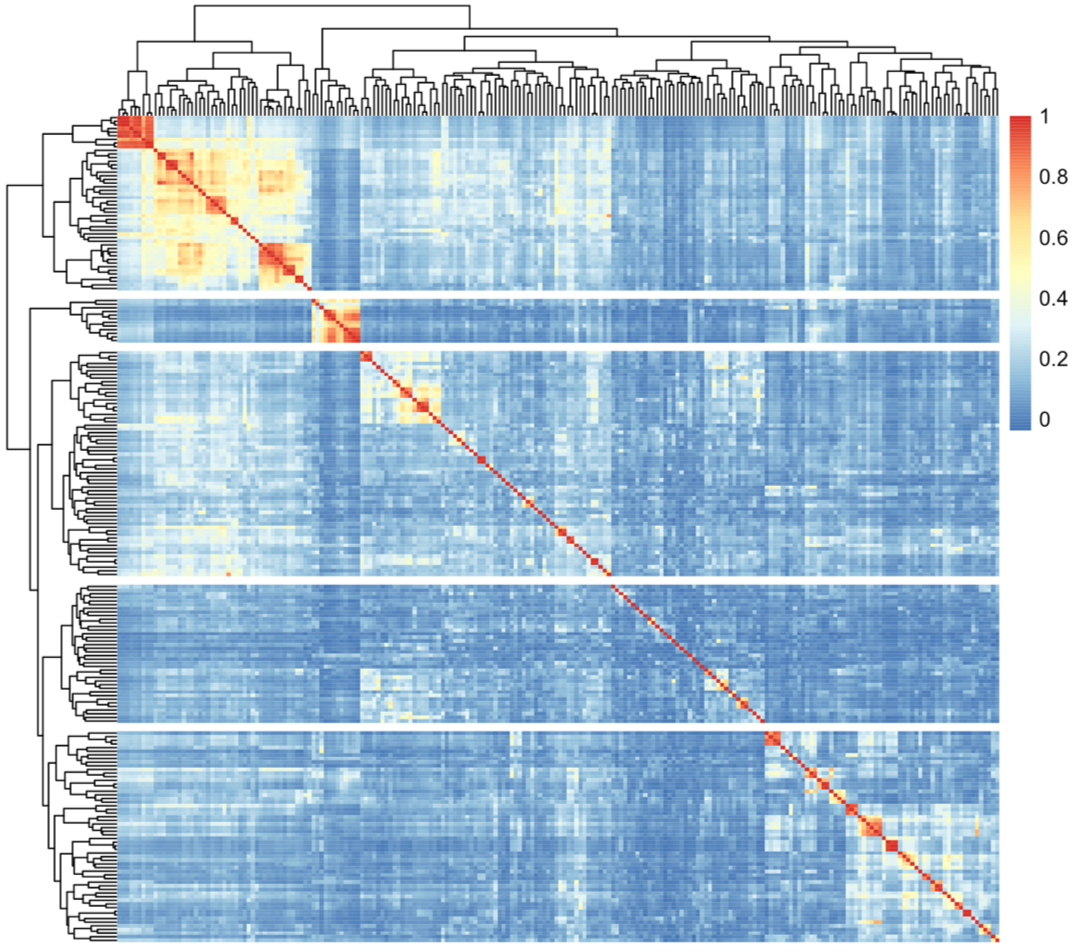
**

**Figure S1. Pairwise feature importance correlation.** The heatmap reports Pearson correlation coefficient values across all protein pairs in the data set. Rows and columns correspond to the 218 proteins (listed in same order). For each protein pair, the color indicates feature importance correlation according to the given continuous spectrum, ranging from no correlation (0; dark blue) to perfect correlation (1, red).

**
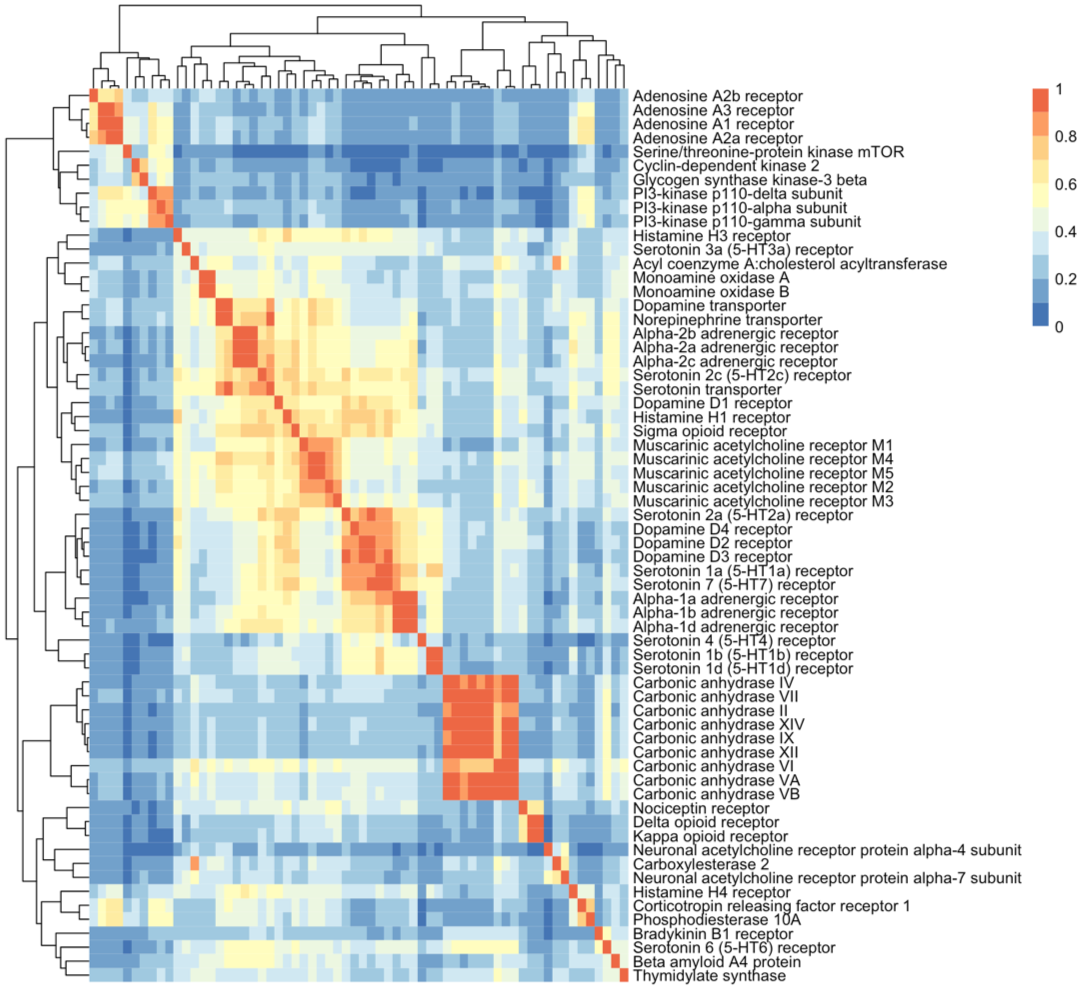
**

**Figure S2. Protein clustering based on feature importance correlation.** The heatmap shows the largest protein cluster in the data set, which is enriched with G protein coupled receptors. Hierarchical clustering was based on pairwise correlation coefficient values. The heatmap reports the feature importance correlation values (color) for pairs of proteins having a Pearson coefficient value of at least 0.5 with a protein in the set. The representation is according to Figure S1, but only contains a subset of proteins.


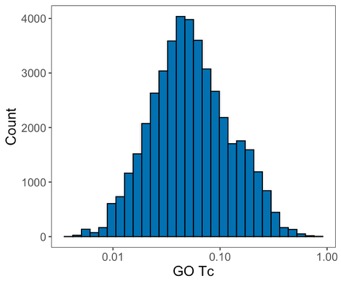


**Figure S3. Tanimoto coefficient distribution for GO terms.** Reported is the distribution of pairwise Tc values for sets of GO terms across proteins in the data set.

**
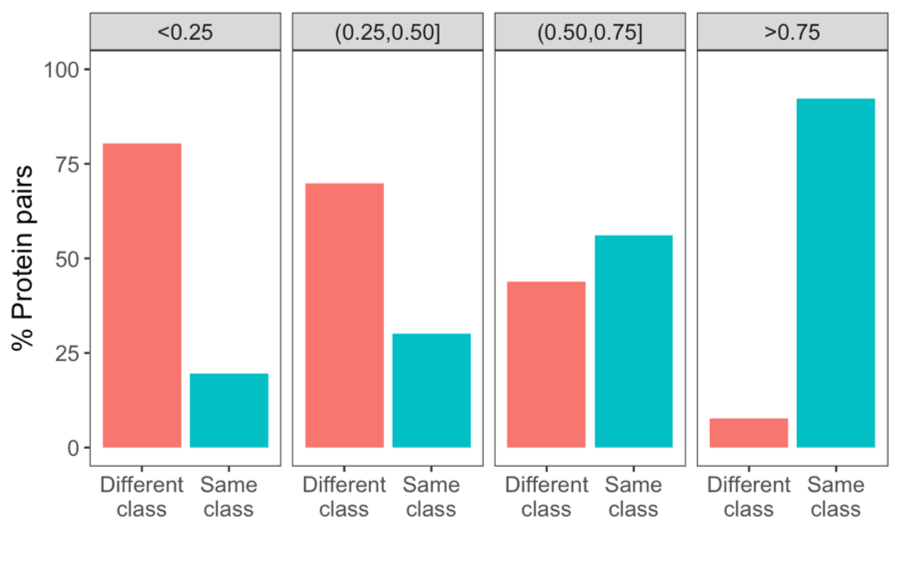
**

**(a)**

**
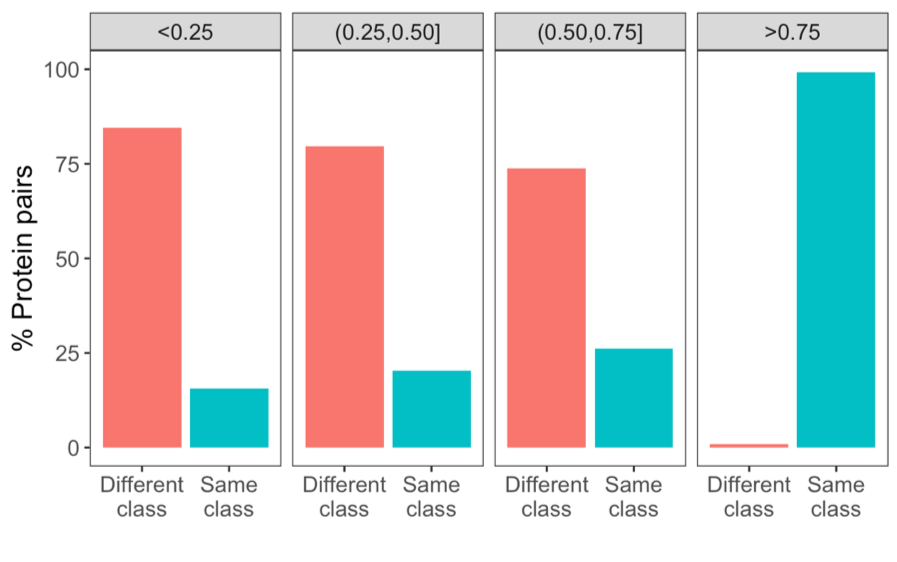
**

**(b)**

**Figure S4. Feature importance correlation and target groups.** The percentage of protein pairs from the same (blue) or different (red) target groups is reported for increasing feature importance correlation values (< 0.25, 0.25-0.50, 0.50-0.75, > 0.75). **(a)** Pearson correlation coefficient. **(b)** Spearman correlation coefficient.
